# Associations between Leisure and Work Time Activity Behavior and 24-h Ambulatory Blood Pressure among Aging Workers

**DOI:** 10.1249/MSS.0000000000003594

**Published:** 2024-11-06

**Authors:** JOOA NORHA, KRISTIN SUORSA, OLLI J. HEINONEN, TEEMU NIIRANEN, KARI K. KALLIOKOSKI, ILKKA H. A. HEINONEN, SARI STENHOLM

**Affiliations:** 1 Turku PET Centre, University of Turku and Turku University Hospital, Turku, FINLAND; 2 Department of Public Health, University of Turku and Turku University Hospital, Turku, FINLAND; 3 Centre for Population Health Research, University of Turku and Turku University Hospital, Turku, FINLAND; 4 Paavo Nurmi Centre and Unit for Health and Physical Activity, University of Turku, Turku, FINLAND; 5 Department of Internal Medicine, Turku University Hospital, University of Turku, Turku, FINLAND; 6 Department of Public Health Solutions, Finnish Institute for Health and Welfare, Helsinki, FINLAND; 7 Research Services, Turku University Hospital and University of Turku, FINLAND

**Keywords:** AMBULATORY BLOOD PRESSURE, LEISURE-TIME, OCCUPATIONAL, PHYSICAL ACTIVITY, SEDENTARY BEHAVIOR

## Abstract

**Purpose:**

The associations between work time, leisure-time, and non-workday physical activity (PA) and sedentary behavior (SED), and 24-h ambulatory blood pressure (BP) are not well known. Therefore, the aim of this study was to evaluate the associations between domain-specific activity behavior and 24-h BP.

**Methods:**

A hundred fifty-six aging workers (mean age, 62.4 (SD 1.0) yr; body mass index, 26.2 (4.5) kg·m^−2^; 84% women; 75% nonmanual occupation) from the Finnish Retirement and Aging study were included. Standing, light and moderate-to-vigorous PA, and SED were measured using thigh-worn accelerometers and work time, leisure-time, and non-workdays were distinguished using a diary. Ambulatory 24-h BP was analyzed as mean daytime and nighttime systolic and diastolic BP, and the nocturnal BP dipping percentage was calculated. Associations were examined with linear regression analysis adjusting for age, sex, occupation, work time mode, job strain, body mass index, BP medication, and accelerometer wear time.

**Results:**

Higher work time SED was associated with lower nighttime diastolic BP (*B* = −0.92; 95% confidence interval (CI), −1.83 to −0.01). In addition, higher work time standing was associated with higher daytime diastolic BP (*B* = 1.34; 95% CI, 0.03 to 2.65), and higher work time light PA was associated with less diastolic BP dipping (*B* = −3.57; 95% CI, −6.80 to −0.34). Moderate-to-vigorous PA in any domain was not associated with ambulatory BP.

**Conclusions:**

Higher work time SED was associated with a more favorable diastolic BP, and higher work time PA was associated with more adverse diastolic BP among aging workers. In conclusion, work time, rather than leisure time or non-workday, activity behavior seems to be associated with 24-h ambulatory BP.

Globally, high blood pressure (BP) causes annually 11 million deaths, and it is rated as the leading modifiable cardiovascular disease risk factor (1). In contrast to conventional home or office BP measurements, 24-h ambulatory BP measurement provides more accurate information on the diurnal BP burden for the cardiovascular system. Indeed, the 24-h ambulatory BP has superior prognostic value over the conventional measures, especially in working adults (2,3).

Based on randomized controlled trials, exercise training (structured and planned physical activity; PA) is effective in lowering ambulatory BP (4). Moreover, preliminary evidence from observational studies suggests that habitual PA, which usually consists of mostly non-exercise PA, that is, every day activities that are not performed for the sole purpose of fitness or health as well as a varying dose of structured exercise training, is associated with a favorable ambulatory BP profile (5,6). Correspondingly, higher sedentary behavior (SED; sitting, reclining, or lying awake activities with <1.5 metabolic equivalent energy consumption) is associated with higher ambulatory systolic BP (5). However, when examining the health consequences of PA and SED, the domain of the activities may play a role in the direction of the association. Specifically, PA at work may in fact be detrimental to cardiovascular health, whereas leisure-time PA has the opposite effect—a phenomenon termed the PA health paradox (7,8). BP burden may be one factor to explain this.

Knowledge on the associations between domain-specific PA and SED and 24-h ambulatory BP is scarce, as, to the best of our knowledge, there are only two studies published: one among 151 Belgian adults with a mean age of 51 yr and diverse occupations (9) and one among 19 American men (mean age, 47 yr) with physically strenuous occupations (10). The former of these reported no associations between device-measured work time PA and systolic BP (diastolic was not reported); however, self-reported heavy lifting was associated with higher systolic BP at work, home, and nighttime, and higher leisure-time PA was associated with lower systolic BP both at work and at home (9). The latter study reported that higher work time light PA (LPA) was associated with higher waking systolic and diastolic BP (10). Furthermore, studies using only office BP measurements have reported conflicting findings: higher occupational PA seems to be associated with lower office BP (11,12), although the evidence is mixed (13,14). Importantly, most of the previous research is conducted among younger or middle-aged workers, and it is therefore important to study these associations also among aging workers who are at a greater absolute risk of cardiovascular disease than younger individuals.

To address these gaps in the literature, the aim of this cross-sectional study was to investigate the association between domain-specific accelerometer-measured activity behavior and 24-h ambulatory BP among aging workers.

## METHODS

This cross-sectional study consists of baseline measurements from the clinical substudy of the Finnish Retirement and Aging study (FIREA) (15). The data for the current analyses were collected before the participants retired between September 2015 and May 2018. A cohort of Finnish-speaking working adults living in Southwest Finland whose estimated retirement date was between 2017 and 2019 were identified from the Finnish pension insurance institute for the municipal sector (Keva) and were invited to participate in the clinical substudy (*n* = 773). Of the invited individuals, 290 agreed to participate. For the current analyses, we included participants who had at least one valid workday accelerometer measurement (with information on work hours) or one valid non-workday and a valid ambulatory BP measurement, leaving 156 included participants. The participant inclusion flowchart is presented in Supplementary Figure 1 (Supplemental Digital Content, http://links.lww.com/MSS/D123).

The study was conducted according to the Declaration of Helsinki, and all participants gave their informed consent before entering the study. The study was approved by the Ethics Committee of the Hospital District of Southwest Finland (84/1801/2014).

### Accelerometry

Activity behavior was measured using Axivity AX3 (Axivity, Newcastle, UK) triaxial accelerometers attached to the right thigh by the study nurse using waterproof adhesive film during the clinical examination visit (16,17). The participants were advised to wear the device for at least 4 d throughout the day and night (at least two working and two non-workdays). For the current analyses, we included participants with at least 1 d of accelerometry in at least one of the domains. The participants were also asked to fill a diary to distinguish sleep, work time, leisure-time (defined as leisure-time on a workday), and non-workdays.

The detailed accelerometry methods and data processing have been described earlier (16,17). In brief, the accelerometer’s raw data were analyzed using ActiPASS (version 0.8) MATLAB program, which is an automatized version of the Acti4 method (18,19). The validated method is specific and sensitive for detecting different activities and body postures in free-living conditions (18,19).

Measurement period was restricted to days between the first and the last date and time recorded in the diary. Sleep time was excluded based on the diary information. Non-wear time was defined as >60 min of no detected movement or periods of 10–60 min where the SDs in the three axes were >0.5 *g* in any second during a 5-s epoch immediately before the period without movement. A valid measurement day was defined as >10 h of measurement. The ActiPASS-detected PA types (18,20) were categorized into four activity types and postures: SED (including sitting and lying, defined as thigh inclination angle >45°), standing, light (LPA; including slow walking and moving), and moderate-to-vigorous PA (MVPA; including fast walking, running, cycling, stair climbing, and other PA). Standing was analyzed separately from LPA and SED, because the thigh-worn device enables this and passive standing (e.g., standing desk work) results in a very low-energy expenditure (21). Moreover, the hemodynamic demands of standing are different from LPA, which requires more lower limb muscle contractions, which in turn has an effect on venous return from the lower extremities.

### Blood pressure

BP was measured from the nondominant arm during a workday over a 24-h period using an oscillometric ambulatory sphygmomanometer (Microlife WatchBP O3, Microlife AG, Widnau, Switzerland), which was attached by the study nurse at the clinical examination visit. As a result, the BP measurement day was the same day as the first day of accelerometry. The device was programmed to measure BP in regular intervals every 30 min. Before each measurement, the device gave an alarm so that the participant could, if possible, stop any activities and sit down with a relaxed arm to ensure successful measurements. If a measurement failed, it was repeated immediately. A diary was used to report the time of going to sleep and waking up. Valid measurement was defined as ≥20 daytime measurements or ≥7 nighttime measurements, as reported previously (22). The outcomes of interest were mean 24 h, daytime (i.e., awake) and nighttime (i.e., asleep) systolic and diastolic BP, and the percentage of nocturnal BP dipping. Nocturnal dipping was calculated as [1 − (nighttime BP/daytime BP)] × 100 (22), and therefore, a higher dipping percentage means larger decrease in BP.

### Covariates

Information on age, sex, and occupation was collected from the pension insurance institute for the municipal sector in Finland (Keva) registry. Occupational status was categorized as manual (e.g., cleaners or maintenance workers) or nonmanual (e.g., teachers or physicians) according to the International Standard Classification of Occupations (classes 5–9 and 1–4, respectively) (23). Work time mode was dichotomized into regular daytime work and other work schedules (e.g., shift work). Job strain was assessed using the Job Content Questionnaire (24) in which the participants evaluated nine items on job control and five items on job demands on five-point scales that ranged from 1 (totally agree) to 5 (totally disagree). Participants with both high demands and low control were categorized as having high job strain, whereas other participants were categorized as having low job strain, as described earlier (22). Body mass index (kg·m^−2^) was calculated from measured weight and height. BP medications (ATC-codes C02, C03, C07, C08, C09, and X01) were inquired by the study nurse (categorized into using BP medication or no BP medication). Self-reported physician-diagnosed hypertension (current and former vs no) was obtained from the questionnaire.

### Statistical methods

Participant characteristics are reported as mean (SD) if not otherwise stated. The associations between SED, standing, LPA, MVPA, and BP variables were analyzed using linear regression analysis. The associations are reported as unstandardized *B* coefficient and 95% confidence interval (CI). The normal distribution of the residuals was evaluated visually, and collinearity was assessed using the variance inflation factors (<5 was considered as no substantial collinearity; no substantial collinearity was present in any of the analyses). The models were adjusted for age, sex, occupation, work time mode, job strain, body mass index, BP medication, and accelerometer wear time.

The statistical analyses were performed in JMP Pro (version 17.0; SAS Institute Inc., Cary, NC).

## RESULTS

### Participants

Participant characteristics are presented in Table 1. Among the included participants (*n* = 156), all had a lower nighttime than daytime BP. The mean number of valid accelerometry days was 2.7 (SD, 1.1) on workdays and 2.3 (0.9) on non-workdays. Seven participants had missing workday accelerometer data, and seven participants had missing non-workday accelerometer data. Characteristics of the included participants, the FIREA clinical substudy participants, and the whole FIREA survey population are presented in Supplementary Table 1 (Supplemental Digital Content, http://links.lww.com/MSS/D123). The current study population was physically more active, had less hypertension, and worked more often in nonmanual and regular day-time occupations in respect of the larger FIREA survey population.

**TABLE 1:** Participant characteristics.

Women, *n* (%)	131 (84)
Age, yr (SD; min–max)	62.4 (1.0; 60–64)
Body mass index, kg·m^−2^	26.2 (4.5)
BP medication, *n* (%)	42 (27)
Smoker, *n* (%)	3 (2)
Nonmanual occupation, *n* (%)	117 (75)
Regular day work, *n* (%)	118 (78)
Low job strain, *n* (%)	134 (88)
Daytime systolic BP, mm Hg	129 (12)
Daytime diastolic BP, mm Hg	78 (7)
Nighttime systolic BP, mm Hg	110 (11)
Nighttime diastolic BP, mm Hg	64 (7)
Nocturnal systolic BP dipping, %	11 (4)
Nocturnal diastolic BP dipping, %	23 (7)
SED at work, h·d^−1^	4.3 (1.6)
SED during leisure, h·d^−1^	5.4 (1.4)
SED non-workday, h·d^−1^	9.3 (1.8)
Standing at work, h·d^−1^	1.8 (1.2)
Standing during leisure, h·d^−1^	1.6 (0.7)
Standing non-workday, h·d^−1^	2.9 (1.1)
LPA at work, h·d^−1^	0.7 (0.5)
LPA during leisure, h·d^−1^	0.8 (0.3)
LPA non-workday, h·d^−1^	1.4 (0.5)
MVPA at work, h·d^−1^	0.6 (0.3)
MVPA during leisure, h·d^−1^	0.8 (0.4)
MVPA non-workday, h·d^−1^	1.2 (0.5)
*N* of accelerometer-measured workdays	2.7 (1.1)
Wear time at work, h·d^−1^	7.4 (1.4)
Wear time during leisure, h·d^−1^	8.6 (1.5)
*N* of accelerometer-measured non-workdays, *n*	2.3 (0.9)
Wear time on non-workdays, h·d^−1^	14.9 (1.2)

Presented as mean (SD) if not otherwise stated.

### Associations between activity behavior and ambulatory BP

The associations between SED and BP measures are presented in Table 2. Work time SED were associated inversely with 24-h and nighttime diastolic BP. There was also an indication of a negative association between work time SED and daytime diastolic BP. Moreover, non-workday SED was positively associated with systolic BP dipping, but not with other BP measures. No statistically significant associations between workday leisure-time SED and BP measures were observed.

**TABLE 2:** Associations between domain-specific SED and 24-h ambulatory BP.

	Work Time		Workday Leisure Time		Non-workday	
	*B* Coefficient	95% CI	*B* Coefficient	95% CI	*B* Coefficient	95% CI
24-h systolic BP	−1.13	−2.73 to 0.46	−0.31	−2.49 to 1.88	−0.004	−1.25 to 1.24
24-h diastolic BP	**−1.02**	**−1.98 to −0.05**	−0.21	−1.53 to 1.11	0.13	−0.63 to 0.89
Daytime systolic BP	−1.10	−2.81 to 0.62	−0.10	−2.43 to 2.24	0.34	−0.98 to 1.66
Daytime diastolic BP	−1.01	−2.05 to 0.03	−0.28	−1.70 to 1.14	0.24	−0.57 to 1.05
Nighttime systolic BP	−1.05	−2.56 to 0.46	−0.46	−2.51 to 1.58	−0.68	−1.87 to 0.51
Nighttime diastolic BP	**−0.92**	**−1.83 to −0.01**	0.002	−1.24 to 1.24	−0.13	−0.85 to 0.59
Systolic BP dipping %	0.17	−0.37 to 0.70	0.21	−0.50 to 0.93	**0.61**	**0.20 to 1.01**
Diastolic BP dipping %	0.47	−0.54 to 1.48	−0.19	−1.54 to 1.15	0.61	−0.17 to 1.40

Statistically significant estimates are bolded.

Adjusted for age, sex, occupation, work time mode, job strain, body mass index, BP medication, and accelerometer wear time.

24 h, all BP measurements over 24 h.

The associations between standing and BP measures are presented in Table 3. Work time standing was associated positively with 24-h and daytime diastolic BP, but not with nighttime diastolic BP or diastolic dipping. No statistically significant associations between workday leisure-time or non-workday standing and BP measures were observed.

**TABLE 3:** Associations between domain-specific standing and 24-h ambulatory BP.

	Work Time		Workday Leisure Time		Non-workday	
	*B* Coefficient	95% CI	*B* Coefficient	95% CI	*B* Coefficient	95% CI
24 h systolic BP	1.37	−0.65 to 3.39	0.57	−2.96 to 4.11	−0.20	−2.13 to 1.73
24-h diastolic BP	**1.29**	**0.07 to 2.51**	0.70	−1.43 to 2.84	−0.01	−1.18 to 1.16
Daytime systolic BP	1.36	−0.80 to 3.53	0.28	−3.49 to 4.05	−0.57	−2.61 to 1.47
Daytime diastolic BP	**1.34**	**0.03 to 2.65**	0.83	−1.46 to 3.12	−0.07	−1.32 to 1.18
Nighttime systolic BP	0.91	−1.00 to 2.83	0.74	−2.56 to 4.05	0.58	−1.26 to 2.43
Nighttime diastolic BP	0.81	−0.35 to 1.98	0.39	−1.62 to 2.39	0.22	−0.90 to 1.34
Systolic BP dipping %	0.03	−0.64 to 0.71	−0.36	−1.51 to 0.80	−0.62	−1.26 to 0.02
Diastolic BP dipping %	0.06	−1.22 to 1.34	0.04	−2.22 to 2.13	−0.58	−1.79 to 0.64

Statistically significant estimates are bolded.

Adjusted for age, sex, occupation, work time mode, job strain, body mass index, BP medication, and accelerometer wear time.

24 h, all BP measurements over 24 h.

The associations between LPA and BP measures are presented in Table 4. Work time LPA was associated inversely with diastolic BP dipping, but not with nighttime or daytime diastolic BP. Moreover, non-workday LPA was inversely associated with systolic and diastolic BP dipping, but not with other BP measures on non-workdays. No statistically significant associations between workday leisure-time LPA and BP were observed.

**TABLE 4:** Associations between domain-specific light PA and 24-h ambulatory BP.

	Work Time		Workday Leisure Time		Non-workday	
	*B* Coefficient	95% CI	*B* Coefficient	95% CI	*B* Coefficient	95% CI
24-h systolic BP	2.70	−2.52 to 7.93	−0.04	−8.19 to 8.10	0.21	−3.65 to 4.07
24-h diastolic BP	1.29	−1.90 to 4.47	−0.30	−5.22 to 4.63	−0.36	−2.70 to 1.99
Daytime systolic BP	2.42	−3.18 to 8.02	−0.56	−9.26 to 8.14	−0.94	−5.03 to 3.14
Daytime diastolic BP	0.96	−2.47 to 4.39	−0.16	−5.45 to 5.13	−0.73	−3.23 to 1.77
Nighttime systolic BP	4.04	−0.87 to 8.94	1.09	−6.53 to 8.71	2.63	−1.04 to 6.30
Nighttime diastolic BP	2.67	−0.31 to 5.65	−0.08	−4.70 to 4.54	0.69	−1.54 to 2.93
Systolic BP dipping %	−1.42	−3.15 to 0.31	−0.93	−3.59 to 1.74	**−2.18**	**−3.42 to −0.94**
Diastolic BP dipping %	**−3.57**	**−6.80 to −0.34**	−0.75	−5.77 to 4.28	**−2.41**	**−4.82 to −0.01**

Statistically significant estimates are bolded.

Adjusted for age, sex, occupation, work time mode, job strain, body mass index, BP medication, and accelerometer wear time.

24 h, all BP measurements over 24 h.

The associations between MVPA and BP measures are presented in Table 5. Work time, workday leisure-time, or non-workday MVPA was not statistically significantly associated with any BP measures. However, work time MVPA tended to be associated with higher daytime and nighttime diastolic BP and less nocturnal systolic and diastolic BP dipping.

**TABLE 5:** Associations between domain-specific moderate-to-vigorous PA and 24-h ambulatory BP.

	Work Time		Workday Leisure Time		Non-workday	
	*B* Coefficient	95% CI	*B* Coefficient	95% CI	*B* Coefficient	95% CI
24-h systolic BP	0.69	−8.05 to 9.43	0.57	−4.96 to 6.10	0.72	−3.34 to 4.77
24-h diastolic BP	2.54	−2.76 to 7.84	−0.26	−3.61 to 3.09	−0.97	−3.43 to 1.49
Daytime systolic BP	0.47	−8.89 to 9.82	0.18	−5.73 to 6.09	−0.04	−4.34 to 4.26
Daytime diastolic BP	2.41	−3.30 to 8.11	−0.15	−3.75 to 3.44	−1.44	−4.06 to 1.18
Nighttime systolic BP	2.79	−5.45 to 11.04	0.65	−4.53 to 5.82	1.68	−2.20 to 5.55
Nighttime diastolic BP	4.69	−0.27 to 9.66	−0.93	−4.06 to 2.21	−0.37	−2.72 to 1.98
Systolic BP dipping %	−1.59	−4.49 to 1.32	−0.05	−1.88 to 1.75	−1.26	−2.60 to 0.09
Diastolic BP dipping %	−5.05	−10.46 to 0.36	1.70	−1.70 to 5.11	−1.28	−3.84 to 1.27

Adjusted for age, sex, occupation, work time mode, job strain, body mass index, BP medication, and accelerometer wear time.

24 h, all BP measurements over 24 h.

## DISCUSSION

In the present study, we report the domain-specific associations between work and leisure-time activity behavior and ambulatory BP. The main findings were that, after adjusting for several potential confounders, 1) SED accumulated during working time associated with a more favorable diastolic BP profile, 2) higher work time standing and LPA were associated with a detrimental diastolic BP profile, and 3) MVPA in any domain was not significantly associated with any of the BP measures. Moreover, the associations between leisure-time or non-workday activity behaviors and BP were mostly nonsignificant, specifically highlighting the importance of occupational activity behavior as a determinant for BP.

Higher work time SED was associated with lower 24-h diastolic BP. In line with our results, the US study of 273 middle-aged desk workers reported that higher occupational SED was associated with lower nighttime diastolic BP (25). Although daytime ambulatory BP was not reported in their study, an inverse association between work time SED and resting diastolic BP was observed (25), which parallels our findings. Physiologically, higher SED leading to lower daytime ambulatory diastolic BP would be logical, as BP is lower at rest than during PA (26). However, higher work time SED associating with lower nighttime diastolic BP is somewhat paradoxical as high SED generally is associated with adverse cardiovascular outcomes (27). As the association was observed only for work time and not leisure-time or non-workday SED, it is possible that the individuals with more sedentary occupations were also higher educated and generally healthier, explaining the lower diastolic BP. We did adjust for job strain and occupation, which in part addresses socioeconomic status, but we did not adjust, for example, for dietary factors, which also have an influence on BP levels.

Higher work time LPA was associated with less diastolic BP dipping. Interestingly, a previous study from the US measuring ambulatory BP on both work and non-workdays in 19 middle-aged men with manual occupations did not observe any associations between work time LPA or MVPA, and nighttime diastolic or systolic BP (10). However, higher work time LPA associated with higher daytime systolic and diastolic BP (10). As both systolic and diastolic BP are of prognostic importance (28), the results together suggest that work time LPA may be detrimentally associated with cardiovascular health. Although not statistically significant, work time MVPA in the present study had a similar-direction trend toward an adverse BP profile, further supporting the detrimental association between work time PA and BP.

In the present study, higher work time standing was associated with higher 24-h diastolic BP, which was mostly driven by daytime diastolic BP. Similarly, although not statistically significant, higher workday leisure-time standing trended toward higher daytime BP (especially diastolic). Furthermore, non-workday standing was not associated with any BP variables. Standing leads to blood pooling in the lower extremities, which in turn leads to lower central blood volume, which is compensated by increased sympathetic activity (e.g., increased cardiac output and vascular tone) (29). Thus, as the ambulatory BP in this study was measured on a workday, it is logical that work time and workday leisure-time standing would be associated with BP, especially with diastolic BP.

To our best knowledge, none of the studies utilizing a 24-h ambulatory BP measurement published to date have reported statistically significant associations between work time MVPA and ambulatory BP (9,10), and our current work further supports this finding. The lack of association may simply be explained by a limited sample size as, in our study, higher work time MVPA trended toward less diastolic dipping (−5.05 (−10.46 to 0.36) percentage points per 1 h of MVPA) and higher nighttime diastolic BP (4.69 (−0.27 to 9.66) mm Hg per 1 h of MVPA), among others. Therefore, a larger sample size may have resulted in statistically significant associations. In addition, most participants in this study had nonmanual occupations. However, as the previous studies have not observed even nonsignificant trends (9,10), more research with larger samples including manual workers is needed to draw conclusions. Moreover, the detrimental effects of occupational PA are partially attributed to the relatively low intensity (e.g., 30% of maximal capacity) and long duration without sufficient recovery time of most PA at work (7), it may be that occupational MVPA and LPA have distinct effects on BP. Speculatively, individuals who have high amounts of occupational MVPA may have higher cardiorespiratory fitness than individuals whose occupational activities are mostly at the level of LPA. Higher fitness could lead to lower BP reactivity during PA and consequently lower ambulatory BP.

The temporality of the 24-h BP measurement and activity behavior measurements should be considered when interpreting the results of this study. BP was measured on the first day of accelerometry, and the BP measurement may have affected activities on this day. However, as the estimates for activity behavior in each domain included more than 1 d (mean of 2.7 workdays and 2.3 non-workdays), the estimates may be interpreted as habitual activity behavior. Similarly, examining the associations between non-workday activities and BP on a workday is of interest, as the risk for cardiovascular disease has been reported to be reduced even when PA is conducted only on 1–2 d of the week (so-called weekend warrior activity pattern) (30). Nevertheless, we did not observe any beneficial associations between non-workday activity behavior and ambulatory BP.

Interestingly, the only statistically significant associations between non-work time PA or SED and ambulatory BP measures were observed for nocturnal BP dipping and non-workday LPA (inverse association, indicating less dipping with more LPA) and non-workday SED (positive association, indicating higher dipping with more SED). The concurrency of these findings is to be expected as SED and LPA often work as counterparts: time not spent in SED is most likely LPA (or standing) and vice versa (31). Speculatively, this finding could be explained by reduced stress and better sleep on non-workdays. The individuals who are more active on non-workdays may be less active on workdays, and therefore, their dipping could be less when measured on a workday, like in this study.

Previous studies on domain-specific SED, PA, and BP with only office BP measurements have reported conflicting results (11–14). Two Danish studies using a cohort of middle-aged manual workers observed that a higher work time step count and reallocating time from SED to PA on work time were associated with lower office BP (11,12). However, among 261 middle-aged Danish workers with varying occupations, higher work time PA was associated with higher diastolic BP (13)—yet, a subsequent Swedish study of over 200,000 male construction workers reported that higher occupational PA was associated with higher systolic and lower diastolic BP but only in the <50-yr-olds (14). Given the mixed results, there is a need for future large scale studies with 24-h ambulatory measurement, as they can provide added prognostic value (2) and give insight to the BP burden over the whole 24 h, and consequently shed light on the role of domain-specific activity behavior on diurnal BP burden.

### Strengths and limitations

Major strengths in this study include the device-based assessment of SED and PA and the use of validated algorithms for the accelerometer data processing (18,19). Moreover, the accelerometry data stratified across domains (i.e., work time, leisure-time, and non-workday) allow the detailed analysis of the distinct domain-specific associations. In addition, the 24-h ambulatory BP measurement provides detailed insight to the BP burden over the whole day and night, and thus, it has been shown to be superior to only office or home BP measurements (2). Finally, the homogeneous age of the participants (i.e., ranging from 60 to 64 yr) increases the study power to detect significant associations, although the generalizability to other age groups remains limited. That said, of the invited 773 individuals, only 290 participated in the clinical substudy, and of them, only about half agreed to undergo an ambulatory BP measurement mostly because the measurement was considered somewhat cumbersome especially during working hours. This, together with the observation that compared with the whole FIREA survey population, the study population was more physically active, had less hypertension, and worked more often in a nonmanual occupation, suggests some healthy participant bias. Therefore, the generalizability among individuals of similar age may also be limited.

A limitation of this study is the cross-sectional setting, which weakens the assessment of causality. However, as BP per se (especially within the non-extreme values) is asymptomatic, it is likely that the observed associations between SED, PA and BP are not due to BP affecting the activity behavior. That said, in addition to underlying cardiovascular health and PA, ambulatory BP is also affected by psychological stress (32), which increases the variability in the BP measurements and consequently potentially decreases the strength of the PA and SED associations. Indeed, individuals with higher job strain and demands have greater BP reduction from workdays to non-workdays (33). Therefore, the analyses in this study were adjusted for job strain. Studying the interactions of physical and psychological stressors on BP at work would be of value in the future, because physically strenuous jobs may also have limited worker control (7). However, ambulatory BP was measured on a workday, and thus, BP on non-workdays remains unknown. Therefore, additionally measuring BP on non-workdays might be valuable in future studies. On the other hand, most days of the week are usually workdays, and consequently, BP measurement on a workday reflects the total BP load better than non-workday measurement (34).

The thigh-worn accelerometers used in this study cannot capture some activity behavior, such as (occupational) lifting or isometric muscle work. Moreover, only 25% of the study participants worked in a manual occupation, and only some of them had a physically very strenuous work. With measures of lifting and isometric work and with more participants in very strenuous occupations, we may have detected more significant associations.

Finally, we used only the diary reports (in contrast to using accelerometry) to define nighttime in the BP measurements. Therefore, it is possible that the participants moved during the night (e.g., walking to the bathroom). However, as a valid nighttime BP required a minimum of seven successful measurements, it is unlikely that the estimate was significantly affected by nighttime activity.

## CONCLUSIONS

In this study, we show that work time, rather than workday leisure time or non-workday, activity behavior is associated with ambulatory BP. Specifically, higher occupational SED is associated with a more favorable diastolic BP profile, whereas occupational higher standing and LPA are associated with a detrimental diastolic BP profile. Large-scale studies with diverse occupations and age groups should be performed to confirm the results and guide future interventions.
